# Gestational Age, Newborn Metabolic Markers and Academic Achievement

**DOI:** 10.3390/ijerph19031549

**Published:** 2022-01-29

**Authors:** George L. Wehby

**Affiliations:** 1 Department of Health Management and Policy, University of Iowa, Iowa City, IA 52242, USA; george-wehby@uiowa.edu; 2 Department of Economics, University of Iowa, Iowa City, IA 52242, USA; 3 Department of Preventive & Community Dentistry, University of Iowa, Iowa City, IA 52242, USA; 4 Public Policy Center, University of Iowa, Iowa City, IA 52242, USA; 5 National Bureau of Economic Research, Cambridge, MA 02138, USA

**Keywords:** school health, gestational age, metabolic health, newborn screening

## Abstract

Background: Gestational age is associated with greater school achievement and variation in newborn metabolic markers. Whether metabolic markers are related to gestational age differences in achievement is unknown. This study examines whether newborn metabolic markers are associated with gestational age differences in performance on standardized school tests. Methods: This retrospective cohort study linked birth certificates of children born in Iowa between 2002 and 2010 to newborn screening records and school tests between 2009 and 2018. The analytical sample includes up to 229,679 children and 973,247 child-grade observations. Regression models estimate the associations between gestational age and 37 newborn metabolic markers with national percentile ranking (NPR) scores on math, reading comprehension, and science tests. Results: An additional gestational week is associated with 0.6 (95% CI: 0.6, 0.7), 0.5 (95% CI: 0.4, 0.5), and 0.4 (95% CI: 0.4, 0.5) higher NPRs on math, reading, and science, respectively. Compared to full term children (37–44 weeks), preterm children (32–36 weeks) have 2.2 (95% CI: −2.6, −1.8), 1.5 (95% CI: −1.9, −1.1), and 1.0 (95% CI: −1.4, −0.7) lower NPRs on math, reading comprehension, and science. Very preterm children (20–31 weeks) have 8.3 (95% CI: −9.4, −7.2), 5.2 (95% CI: −6.2, −4.0), and 4.7 (95% CI: −5.6, −3.8) lower NPRs than full term children on math, reading, and science. Metabolic markers are associated with 27%, 36%, and 45% of gestational age differences in math, reading, and science scores, respectively, and over half of the difference in test scores between preterm or very preterm and full term children. Conclusions: Newborn metabolic markers are strongly related to gestational age differences in school test scores, suggesting that early metabolic differences are important markers of long-term child development.

## 1. Introduction

Gestational age is positively associated with children’s academic performance [1], and preterm children have lower achievement on average than full-term children [2,3,4], but also more risk for early neurodevelopmental and learning problems [5,6]. Gestational age is correlated with levels of newborn markers that are used to screen for rare metabolic and endocrine problems that can be seriously detrimental to child development or even fatal if undetected and untreated. Markers for congenital hypothyroidism, adrenal hyperplasia, and amino acid and fatty acid metabolism disorders are strongly correlated with gestational age (joint correlation coefficient of about 0.5) and can be used to predict preterm birth status with reasonable precision [7,8,9]. This correlation between newborn metabolic markers and gestational age suggests that these markers captures important differences in fetal development for which they are not the primary measure. It also suggests possible linkages to long-term developmental outcomes such as academic achievement.

Despite the clinical and public health importance of understanding connections between newborn metabolic markers throughout their entire ranges including outside of thresholds clinically indicative of metabolic problems, there is little direct empirical evidence, especially from large-scale population-based studies. One study from Australia reported an association between differences in neonatal thyroid stimulating hormone (TSH) levels below the clinically used threshold for hypothyroidism and numeracy test scores [10]. That study, which only analyzed TSH levels, suggests that newborn markers in ranges outside clinically used thresholds to screen for metabolic disorders are broadly relevant for child development. To our knowledge, no study has evaluated associations between multiple newborn metabolic markers throughout their entire ranges, gestational age, and child achievement.

This study examines the associations between gestational age and academic achievement measured via standardized school tests and the extent to which metabolic markers are related to gestational age differences in achievement. The study uses unique population-based data linkages in Iowa including birth certificates, school tests, and newborn screening program data.

## 2. Methods

### 2.1. Population and Sample

The study includes children born in Iowa between 2002 and 2010 who completed newborn screening and attended elementary school in Iowa between school years 2009/2010 and 2017/2018. The birth period begins in 2002 because this is the first year when the newborn screening marker panel was expanded to include multiple amino acid and fatty acid markers associated with gestational age. The study sample is from a unique linkage of three datasets: birth certificate data from the Iowa Department of Public Health, newborn screening data from the Iowa State Hygienic Laboratory, and standardized school test scores from the Iowa Testing Programs. The birth certificate data were independently matched to school tests based on child’s name and date of birth, and to the newborn screening data based on combinations of child’s name, date and time of birth, and sex, birth hospital, and mother’s name and date of birth. Between 2002 and 2010, there were 344,578 children born in Iowa based on birth certificates. Of those, 334,873 children (97.2% of population) were matched to newborn screening data. Of the matched children, 322,854 children had complete data on all 37 metabolic markers; of those, 320,395 (99.2%) had complete data on gestational age and demographic control variables from the birth certificate data.

Of the sample with matched and complete birth certificate and metabolic marker data (*n* = 320,395), 229,863 (71.7%) matched to at least one school test. Because testing increases with age (grade), only 18% of children born in 2010 and 57% of children born in 2009 were matched to a school test, compared to 82% of children born in 2002–2008. In sensitivity checks (discussed below), children born in 2009–2010 are excluded. In total, 67% of children born in Iowa between 2002 and 2010 (study population) were matched across the three datasets and had complete data on all metabolic markers, included birth certificate variables, and at least one test score between 1st grade and 10th grade. Comparisons between the included and excluded samples are discussed below.

The gestational age measure is the obstetric estimate of gestational age recorded in birth certificates (in weeks). Infants born at gestational age below 20 weeks or above 44 weeks are excluded to reduce the chance of recording errors. Gestational age in weeks and indicators for preterm birth (<37 weeks), preterm but not very preterm (32–36 weeks), and very preterm (20–31 weeks) birth, with full term birth (37–44 weeks) are examined.

The newborn screening data includes 37 metabolic markers previously shown to correlate with gestational age [7]. These markers include an enzyme for galactosemia, a hormone for congenital adrenal hyperplasia, another hormone for congenital hypothyroidism, 7 amino acids for amino acidemias and urea cycle disorders, and 27 acylcarnitines for fatty acid oxidation disorders (detailed list in Supplementary Table S1 online). These markers were measured as part of Iowa’s newborn screening program by the State Hygienic Laboratory using hormone testing and tandem mass spectrometry [11]. Newborn screening tests that were repeats (about 5% of tests) or those rejected by the screening program due to poor quality (around 1% of tests) are excluded.

The academic achievement data are from the Iowa Tests of Basic Skills, the Iowa Tests of Educational Development, and Iowa Assessments. These tests are nationally recognized, standardized, and highly valid and reliable achievement tests that cover major academic subject domains. They have strong psychometrics and were standardized using a nationally representative student sample [12,13]. The tests are administered to virtually all students in Iowa schools (public and private schools) and have been among the most commonly used school tests of academic achievement nationwide. They have also been successfully included in prior research studies linking them to birth certificate data [14,15,16]. Tests included in this study are for elementary grades 1–5, middle school grades 6–8, and high school grades 9–10. Test scores on mathematics, reading comprehension, and science standardized as national percentile rankings are included.

The study sample includes observations with complete data on all included birth certificate variables (described below), all 37 metabolic markers, and at least one test score on one test domain. From the sample of 229,863 children with complete data on birth certificate and newborn screen variables who were matched to at least one school test, the analytical sample ranges between 222,439 and 229,679 unique children and between 909,454 and 973,247 child-grade observations depending on test domain (since some children were not tested on all three domains at a given grade). In general, science tests were less frequently administered at early grades than math and reading. The study was reviewed and approved by the University of Iowa Institutional Review Board.

### 2.2. Statistical Analysis

Regression analysis is employed to estimate: 1—the association between metabolic markers and gestational age; 2—the associations between gestational age and school test scores; and 3—the extent to which metabolic markers statistically account for the associations between gestational age and test scores. Three measures of gestational age are used. The first is a continuous measure of gestational age in weeks, which captures the whole variation in gestational age and allows estimating the association of an additional gestational week with test scores. Two indicators for preterm birth are also included to understand these associations specifically for preterm children. One measure is, a 0/1 indicator for preterm birth < 37 weeks, and another is 0/1 indicator very preterm birth < 32 weeks. To examine the association between metabolic markers and gestational age, three regression specifications are estimated. The first model regresses the three gestational age measures, one at a time, on sociodemographic variables as follows:

(1) $$GestationalAge_{i}=X_{i}+e_{i}$$

For child *i*, *GestationalAge* is one of the three gestational age measures mentioned above. *X* includes the following variables from birth certificates: child’s gender and birth year 0/1 indicators, and maternal race, age, marital status, and education. In all equations (1 through 6 below), the last term designates the error (residual term).

Next, the 37 metabolic markers are added as covariates to this regression to examine the change in the R-squared compared to the first regression. That change in R-squared gives the association between these metabolic markers jointly and gestational age. First, the levels of metabolic markers (*MetabolicMarkers*) are added. Then, in a third regression, the metabolic marker squares and cubic terms to account for possible non-linearity in their associations with gestational age.

(2) $$GestationalAge_{i}=X_{i}+MetabolicMarkers_{i}+u_{i}$$

(3) $$\begin{array}{l} GestationalAge_{i}=X_{i}+MetabolicMarkers_{i}+MetabolicMarkers\_Squared_{i}+\\ \;\;\;\;\;\;\;\;MetabolicMarkers\_Cubic_{i}+v_{i} \end{array}$$

Equations (1) through (3) are estimated for the analytical sample with complete data on all included sociodemographic and metabolic markers, and math scores (229,679 children). These models are also estimated for children (*n* = 90,532, or 78.9% of the 114,715 children not included in the analysis) with complete data on sociodemographic and metabolic markers who were not matched to school tests to check for any sample selection bias from not matching to school tests.

Next, the association between gestational age and academic achievement is estimated for the analytical sample with complete and linked data from the three datasets (birth certificates, newborn screening, and school tests) as follows:

(4) $$TestScore_{it}=\beta_{1}.GestationalAge_{i}+X_{i}+Grade_{it}+Year_{t}+r_{it}$$

For child *i* tested in school year *t*, *TestScore* is the child’s score on one of the three test domains (mathematics, reading comprehension, and science). *Grade* includes fixed effects (0/1 indicators) for grade level, and *Year* includes fixed effects (0/1 indicators) for school year. *β_1_* is the estimated association between gestational age and test scores. One model is estimated including gestational age in weeks, and another model including two indicators in *GestationalAge* for preterm but not very preterm (32–36 weeks), and very preterm (20–31 weeks), with full term birth (≥37 weeks) as the reference category.

Next, the extent to which metabolic markers statistically account for the association between gestational age and school test scores is examined by adding these markers as covariates into the test score regression. Model (5) adds the levels of metabolic markers, while model (6) also adds their squared and cubic terms.

(5) $$TestScore_{it}=\beta_{2}.GestationalAge_{i}+MetabolicMarkers_{i}+X_{i}+Grade_{it}+Year_{t}+\omega_{it}$$

(6) $$\begin{array}{l} TestScore_{it}=\beta_{3}.GestationalAge_{i}+MetabolicMarkers_{i}+MetabolicMarkers\_Squared_{i}+\\ \;\;\;\;\;\;\;MetabolicMarkers\_Cubic_{i}+X_{i}+Grade_{it}+Year_{t}+k_{it} \end{array}$$

*β*_2_ is the estimated association between each of the gestational age measures and test scores adjusting for metabolic marker levels (*β*_3_ is the same adjusting for non-linearity in metabolic marker effects). Then, *β*_2_ and *β*_3_ are compared with *β*_1_ to infer the extent to which metabolic markers are related to the association between gestational age and test scores; the difference from *β*_1_ is the part of association between gestational age and tests scores statistically accounted for by metabolic markers. All models are estimated using ordinary least squares. In models 4 through 6 with child-grade observations, standard errors are clustered at the child level and are robust to general heteroscedasticity.

### 2.3. Sensitivity Checks

Additional models are estimated to evaluate the sensitivity of estimates to certain sample and model choices. The first check estimates the association between gestational age and school test scores excluding children with gestational age within 42–44 weeks to avoid possible data errors. The second sensitivity check estimates this association excluding children born in 2009–2010 since school testing is less common at early grades. Finally, as a more flexible way to account for possible non-linearity in the metabolic marker effects than adding their cubic and squared terms, the model includes instead 0/1 indicators for their 5-percentile ranges.

## 3. Results

### 3.1. Sample Description

Table 1 shows summary statistics for the child-grade analytical sample. About 91% were full-term, 8% preterm (32–36 weeks), and 1% very preterm (20–31 weeks). Forty nine percent were females. The majority of mothers were White (94%), 3% were Blacks, and 3% were of other race. At birth, average maternal age was 27 years and 70% of mothers were married. Nearly 13% of mothers had not graduated high school, 26% graduated high school, 32% attended but had not graduated from college, and 30% were college graduates. Average NPR scores were 60 for math and reading comprehension and 59 for science.

Table 2 shows summary statistics for gestational age and demographic variables for the excluded sample of births due to not matching or incomplete data (*n* = 114,715) compared to the sample of children with complete data on birth certificate and newborn screen variables and at least one school test (*n* = 229,863). Average gestational age was comparable between the two samples (38.5 versus 38.7), but preterm (32–36 weeks) and very preterm (20–31) birth rates were higher in the excluded sample (8.5% versus 7.8%, and 2.1 versus 1.0%, respectively). The excluded sample had a higher proportion of minority mothers (13.3% versus 7.7%), mothers who had not completed high school (17.5% versus 13.3%), and single mothers (35.9% versus 31.7%).

### 3.2. Correlation between Metabolic Markers and Gestational Age

Table 3 shows the correlation of gestational age measures with metabolic markers (based on models 1 and 2 above) for the analytical sample with complete data on all included demographic and metabolite variables, and math scores (*n* = 229,679 children). Results when adding squared and cubic terms of the metabolic markers (model 3) are in Supplementary Table S2 online. Metabolic markers statistically account for 44% of the variation in continuous gestational age, and 47% when adding squared and cubic terms. They also account for about a third of the variation in preterm (<37 weeks) and very preterm (<32 weeks) birth. Supplementary Table S3 online shows overall similar correlations between metabolic markers and gestational age for children with complete data on demographic and metabolite variables but not matched to school tests (*n* = 90,532).

### 3.3. Association of Gestational Age and School Test Scores

Table 4 reports the associations between gestational age and school test scores estimated from model (4). Longer gestational age is associated with higher math, reading comprehension, and science scores by 0.6, 0.5, and 0.4 NPRs, respectively, or by 0.02 standard deviations (SDs) of these scores. Preterm children (32–36 weeks) have lower scores than full term children (37–44 weeks) by 2.2, 1.5, and 1.0 NPRs on math, reading comprehension, and science, or by 0.08, 0.05, and 0.04 SDs, respectively. Very preterm children (20–31 weeks) have lower scores by 8.3, 5.1, and 4.7 NPRs on math, reading, and science, respectively, or by 0.3 SD for math and 0.2 SD for reading and science.

### 3.4. Association of Metabolic Markers with Gestational Age Differences in School Test Scores

**Figure 1:**
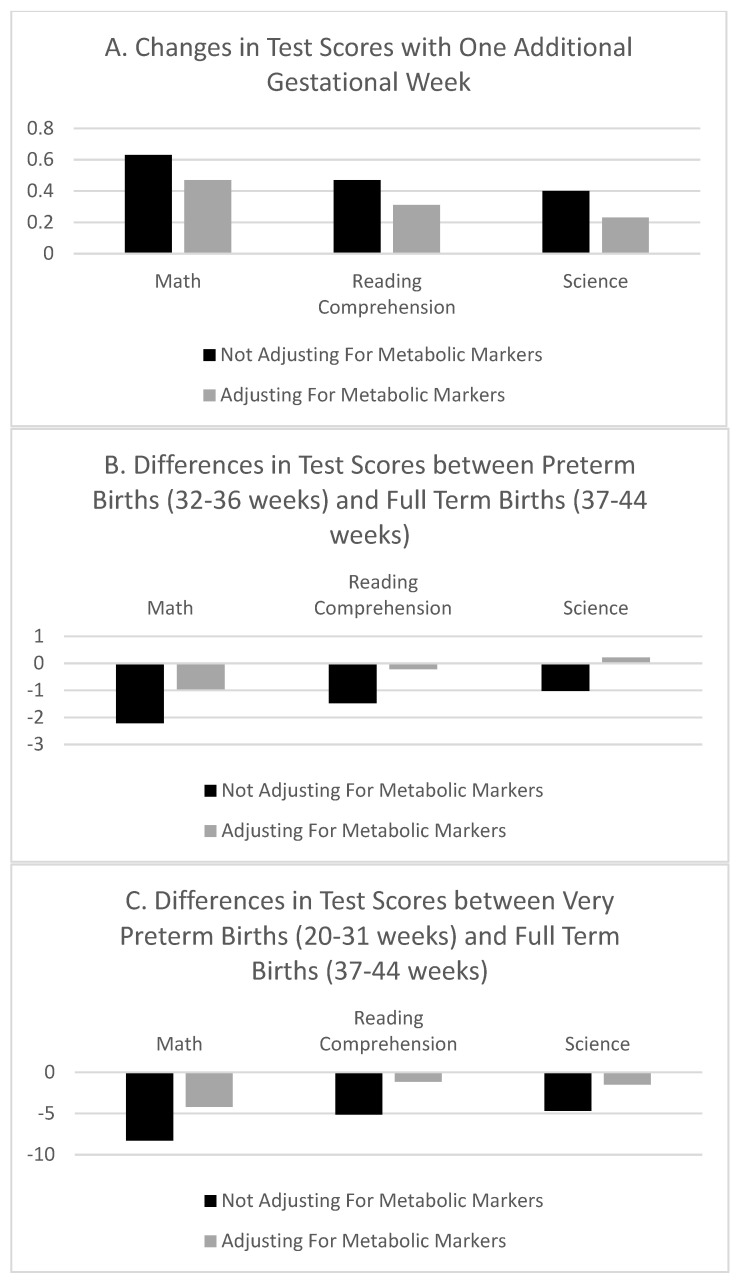
Differences in Test Scores by Gestational Age with and without Adjustment for Metabolic Markers. Notes: The figure reports the regression coefficients of gestational age measures in regressions of school tests scores as the dependent variables (outcomes) on gestational age and covariates. (**A**) is for gestational age in weeks. (**B**) is comparing preterm (32–36 weeks) and full term (37–44) births. (**C**) is comparing very preterm (20–31 weeks) and full term (37–44) births. The unit of analysis is a child-grade observation. A separate regression model is estimated for each bar using ordinary least squares. Standard errors are clustered at the child level. Academic test scores are national percentile rankings. Tests from 1st through 10th grade levels are included for school years 2009–2010 through 2017–2018. All models adjust for gender, maternal race, age, marital status, and education, as well as child’s birth year fixed effects, grade fixed effects, and school year fixed effects. Additional models adjust for metabolic markers. Sample limited to children born between 2002 and 2010.

Table 5 reports the associations between gestational age measures and school test scores adjusting for metabolic marker levels; results adding the squared and cubic terms of metabolic markers are in Supplementary Table S4 online. Figure 1 plots estimates of gestational age associations with test scores with and without adjustment for metabolic markers. Adjusting for metabolic markers substantially reduces the association between gestational age in weeks and test scores, with little difference between adding the squared and cubic terms of metabolic markers or just their levels. Further, adjusting for metabolic markers eliminates most of the differences in test scores between preterm or very preterm and full term children. Specifically, adjusting for metabolic markers reduces the associations between gestational age in weeks and math, reading, and science scores by 27%, 36%, and 45%, respectively. It also reduces the difference in math scores between preterm and full term children by 57%, and practically eliminates the difference in reading and science. Similarly, this adjustment reduces the difference in math scores between very preterm and full term children by 51%, and their difference in reading and science by 68%.

### 3.5. Sensitivity Checks

There are similar associations between gestational age and school test scores when excluding gestational age above 41 weeks (Supplementary Table S5 online) and children born in 2009–2010 (Supplementary Table S6 online). Furthermore, results are comparable when controlling for 5-percentile increments of metabolic marker levels (Supplementary Table S7 online).

## 4. Discussion

Gestational age is associated with academic achievement during childhood but also with early life metabolic markers. This study fills a major knowledge gap in the literature about the extent to which newborn metabolic markers are related to the association between gestational age and school test scores using a unique linkage of birth certificates, standardized school test scores, and newborn screening program for children from Iowa. Children born very preterm and preterm have lower scores than full-term infants by 0.2–0.3 SDs and 0.04–0.08 SDs, respectively, with larger differences for math than reading and science. Adjusting for newborn metabolic markers substantially reduces the association between gestational age and school test scores. For continuous gestational age, this adjustment reduces this association by one third (for math) to half (for science). Moreover, adjusting for metabolic markers reduces the difference in math scores between preterm or very preterm and full-term children by half, and nearly eliminates the difference in reading and science. To our knowledge, this is the first evidence on the relevance of multiple metabolic markers examined jointly for academic achievement using large-scale population-based data.

The study includes 37 markers related to galactosemia, congenital hypothyroidism, adrenal hyperplasia, and amino acid and fatty acid metabolism disorders that statistically account for nearly half of the variation in gestational age. The levels of such endocrine and metabolic markers may reflect differences in fetal growth of certain body organs but also stress and developmental issues and pregnancy complications and maternal health. The evidence from this study suggests that these markers are broadly connected with fetal and child development throughout their entire ranges including levels considered normal and outside clinically used thresholds to indicate metabolic disorders. In that way, they might serve as early markers of long-term child development. This evidence also suggests that the relationship between gestational age and long-term development depends partly on early life endocrine and metabolic markers. Understanding the associations between newborn metabolic markers and achievement across gestational age ranges (including among preterm and full term children) and among children of the same gestational age warrants further investigation. Building on this work with additional research on the possible connections between neonatal metabolic markers and long-term child development outcomes could ultimately offer important knowledge on the role of specific markers and the information they might provide on developmental risks and the opportunities to address these risks early on.

The study findings on the correlation of gestational age measures with metabolic markers are consistent with those from previous studies including a study from the same population but fewer birth years. The results on the associations between gestational age and school test scores are also consistent with the literature on this question and are similar to those from another population-based study of children in Florida [4]. This consistency supports the study results on these associations. To our knowledge, no previous study has examined changes in the association between gestational age and achievement outcomes after adjusting for the metabolic markers this evaluate, so those results cannot be compared to previous studies.

A key strength of this study is linking three population-based datasets (birth certificates, newborn screening, and standardized school tests scores) with data on gestational age and demographic variables (birth certificates), multiple newborn metabolic markers, and standardized school tests on three areas (math, reading, and science). The study has three limitations. First, the study only includes metabolic markers previously connected with gestational age in this population. It is possible that other metabolic markers can further explain the remaining difference in test scores by gestational age. Second, the data is from one state with little racial/ethnic variation (majority of population are non-Hispanic Whites); understanding the generalizability of findings in more diverse populations is important. Third, the analytical sample is approximately two thirds of the total population mostly due to not matching to school tests. The excluded sample has higher rates of very preterm and preterm births, which could elevate neonatal and infant mortality and developmental problems during childhood that may affect school attendance, particularly for very preterm birth [17,18]. However, in nearly 80% of the excluded sample with complete data on metabolic markers, these markers are similarly associated gestational age as in the analytical sample, suggesting little sample selection bias in non-matching affecting the association between metabolic markers and gestational age. Furthermore, the higher proportions of very preterm and preterm infants in the excluded sample suggests that if anything, the association between gestational age and achievement would be underestimated. Finally, as noted above, differences in test scores by gestational age in this study are similar to another study of children in Florida [4], which is another indicator of little impact of sample selection on the estimates.

## 5. Conclusions

Newborn metabolic markers are associated with over half of the difference in school math scores between preterm or very preterm and full-term children and nearly the full difference in reading and science scores. This evidence from a population-based linkage of birth certificates, standardized school test scores, and newborn screening program data suggests that differences in newborn metabolic levels including in ranges considered normal and not indicative of metabolic disorders are important markers of long-term child development. Examining patterns of variation across various metabolic markers is an important future research direction.

## Figures and Tables

**Table 1:** Summary statistics of analytical sample of child-grade observations.

Variable	Mean (SD) or # (%)
Gestational Age (weeks), Mean (SD)	38.7 (1.9)
Categorical gestational age, # (%)	
Very preterm (20–31 weeks)	9607 (1.0)
Preterm (32–36 weeks)	75,017 (7.7)
Full term (≥37 weeks)	888,623 (91.3)
Child’s sex, # (%)	
Female	481,046 (49.4)
Male	492,201 (50.6)
Maternal race, # (%)	
White	911,538 (93.7)
Black	29,043 (3.0)
Other	32,666 (3.4)
Maternal age (years), Mean (SD)	27.3 (5.6)
Maternal marital status, # (%)	
Married	679,146 (69.8)
Not married	294,101 (30.2)
Maternal Education, # (%)	
Less than high school	124,814 (12.8)
High school	252,195 (25.9)
Some college	308,750 (31.7)
College graduate	287,488 (29.5)
Math score, Mean (SD)	59.5 (27.5)
Reading comprehension score, Mean (SD)	60.3 (28.6)
Science score, Mean (SD)	59.3 (25.2)

Notes: The descriptive statistics for math scores and all demographic variables are based on the analytical sample of child-grade observations with complete data on all those variables and metabolic variables (*n* = 973,247). The descriptive statistics for reading and science scores are based on the samples with complete data on these scores (*n* = 973,353 and *n* = 909,454 child-grade observations, respectively) and demographic variables and metabolic marker variables. The test scores are in national percentile rankings, which is the ranking of the child on a national distribution of these scores. The demographic variables and gestational age are from birth certificates. SD = standard deviation.

**Table 2:** Descriptive statistics for sample excluded from analysis due to not matching or incomplete data compared to included sample.

Variable	Sample Excluded from Analysis Mean (SD) or # (%)	Included Sample Mean (SD) or # (%)
Gestational age (weeks), Mean (SD)	38.5 (2.4)	38.7 (1.9)
Categorical gestational age, # (%)		
Very preterm (20–31 weeks)	2368 (2.1)	2314 (1.0)
Preterm (32–36 weeks)	9738 (8.5)	17,861(7.8)
Full term (≥37 weeks)	102,609 (89.5)	209,688 (91.2)
Child’s sex, # (%)		
Female	55,179 (48.1)	112,962 (49.1)
Male	59,535 (51.9)	116,901 (50.9)
Maternal race, # (%)		
White	98,529 (86.5)	212,113 (92.3)
Black	6227 (5.5)	7558 (3.3)
Other	9143 (8.0)	10,192 (4.4)
Maternal age (years), Mean (SD)	27.0 (5.7)	27.2 (5.6)
Maternal marital status, # (%)		
Married	73,532 (64.1)	156,940 (68.3)
Not married	41,123 (35.9)	72,923 (31.7)
Maternal Education		
Less than high school	19,820 (17.5)	30,549 (13.3)
High school	25,811 (22.8)	56,780 (24.7)
Some college	34,187 (30.2)	74,832 (32.6)
College graduate	33,498 (29.6)	67,702 (29.5)

Notes: The sample excluded from the analytical sample due to not matching or incomplete data includes 114,715 children (born in Iowa over the same period of 2002–2010). The “Included Sample” in this table includes 229,863 unique children with complete data on gestational, other included birth certificate variables, all metabolic variables, and at least one test score (on any of the three test domains) between 1st and 10th grade. As noted in the paper and shown in Table 3, t the number of observations in the analytical sample in regressions for the three test domains ranges between for All variables included in this table are from birth certificates. The excluded sample includes children born in Iowa over the same period (2002–2010) who are not included in the analysis due to not matching or due to incomplete data on some variables. The counts of excluded children for some variables may not add to 114,715 due to missing data. SD = standard deviation.

**Table 3:** Variation in gestational age associated with metabolic markers.

Gestational Age Measure	Metabolic Markers Levels	Metabolic Markers Levels, Squared Terms and Cubic Terms
Gestational age in weeks	44.0%	47.3%
Preterm birth (<37 weeks)	29.5%	32.8%
Very preterm birth (<32 weeks)	25.4%	31.3%
Number of children	229,679	229,679

Notes: The unit of analysis is a child. A separate regression model is estimated by regressing each gestational age measure on gender, maternal race, age, marital status, and education, and child’s birth year fixed effects first without metabolic markers and then adding them as covariates. Percent of variation in gestational age associated with metabolic markers is the change in R-squared after adding metabolic markers as covariates. The sample includes children with at least one math test. Sample limited to children born between 2002 and 2010.

**Table 4:** Associations between gestational age measures and school test scores without adjusting for metabolic markers.

Gestational Age Measure	Math	Reading Comprehension	Science
Panel A: Gestational age in weeks	0.63 ***	0.47 ***	0.40 ***
	(0.03) [0.58, 0.69]	(0.03) [0.41, 0.53]	(0.03) [0.35, 0.45]
Panel B: Preterm or Very Preterm (versus Full term)			
Preterm Birth 32–36 weeks	−2.22 ***	−1.48 ***	−1.02 ***
	(0.20) [−2.60, −1.83]	(0.21) [−1.88, −1.07]	(0.17) [−1.36, −0.67]
Very Preterm Birth < 32 weeks	−8.28 ***	−5.13 ***	−4.70 ***
	(0.54) [−9.35, −7.22]	(0.57) [−6.24, −4.02]	(0.48) [−5.63, −3.76]
Number of child-grade observations	973,247	973,353	909,454
Number of unique children	229,679	229,521	222,439

Notes: The table reports the regression coefficients of gestational age measures in regressions of school tests scores as the dependent variables (outcomes) on gestational age and covariates. Standard errors are in parentheses, and 95% confidence intervals are in brackets. The unit of analysis is a child-grade observation. A separate regression model is estimated for each panel and academic testing domain using ordinary least squares. The reference category in panels B is gestational age ≥ 37 weeks. Standard errors are clustered at the child level. Academic test scores are national percentile rankings. Tests from 1st through 10th grade levels are included for school years 2009–2010 through 2017–2018. All models adjust for gender, maternal race, age, marital status, and education, as well as child’s birth year fixed effects, grade fixed effects, and school year fixed effects. Sample limited to children born between 2002 and 2010. *** *p* < 0.01.

**Table 5:** Associations between Gestational Age Measures and School Test Scores Adjusting for Metabolic Markers.

Gestational Age Measure	Adjusting for Levels of Metabolic Markers		
	Math	Reading Comprehension	Science
Panel A: Gestational age in weeks	0.47 ***	0.31 ***	0.23 ***
	(0.04) [0.40, 0.54]	(0.04) [0.24, 0.39]	(0.03) [0.17, 0.30]
Panel B: Preterm or Very Preterm (versus Full term)			
Preterm Birth 32–36 weeks	−0.96 ***	−0.22	0.21
	(0.22)	(0.23)	(0.20)
	[−1.39, −0.52]	[−0.67, 0.24]	[−0.17, 0.60]
Very Preterm Birth < 32 weeks	−4.21 ***	−1.61 **	−1.50 ***
	(0.63)	(0.67)	(0.56)
	[−5.45, −2.97]	[−2.91, −0.30]	[−2.60, −0.41]
Number of child-grade observations	973,247	973,353	909,454
Number of unique children	229,679	229,521	222,439

Notes: The table reports the regression coefficients of gestational age measures in regressions of school tests scores as the dependent variables (outcomes) on gestational age and covariates. Standard errors are in parentheses, and 95% confidence intervals are in brackets. The unit of analysis is a child-grade observation. A separate regression model is estimated for each panel and academic testing domain using ordinary least squares. The reference category in panels B is gestational age ≥ 37 weeks. Standard errors are clustered at the child level. Academic test scores are national percentile rankings. Tests from 1st through 10th grade levels are included for school years 2009–2010 through 2017–2018. All models adjust for gender, maternal race, age, marital status, and education, as well as child’s birth year fixed effects, grade fixed effects, school year fixed effects, and metabolic markers. Sample limited to children born between 2002 and 2010. ** *p* < 0.05, *** *p* < 0.01.

## Data Availability

The study uses data from the Iowa Department of Public Health and from the Iowa Testing Programs. Data access requires applications to the data providers.

## Supplementary Materials

The following supporting information can be downloaded at: https://www.mdpi.com/article/10.3390/ijerph19031549/s1, Supplementary Table S1: Metabolic Markers from Newborn Screening Program Included in the Study, Supplementary Table S2: Variation in Gestational Age Associated with Metabolic Markers and Their Squared and Cubic Terms, Supplementary Table S3: Variation in Gestational Age Associated with Metabolic Markers in Sample Not Matched to School Tests, Supplementary Table S4: Variation in Gestational Age Associated with Metabolic Markers in Sample Not Matched to School Tests, Supplementary Table S5: Associations between Gestational Age Measures and School Test Scores Excluding Gestational Ages above 41 Weeks and without Adjusting for Metabolic Markers, Supplementary Table S6: Associations between Gestational Age Measures and School Test Scores Excluding Birth Years 2009 and 2010, Supplementary Table S7: Associations between Gestational Age Measures and School Test Scores Adjusting for 5-Percentile Ranges of Metabolic Markers.
