# Supplementary material for: Gestational Age, Newborn Metabolic Markers and Academic Achievement
Source: Int J Environ Res Public Health. 2022 Jan 29;19(3):1549. doi: 10.3390/ijerph19031549 (PMC8834716; doi:10.3390/ijerph19031549)
Supplement: Supplementary file 1 [file ijerph-19-01549-s001.zip › ijerph-1514571-supplementary.pdf]

Supplementary Tables Online

Supplementary Table S1. Metabolic Markers from Newborn Screening Program Included in the Study

**Enzymes**	**Acylcarnitines**	**Acylcarnitines**
Galactose-1-Phosphate Uridyl Transferase (GALT)	Acetylcarnitine (C2)	Decenoylcarnitine (C10:1)
**Hormones**	Propionylcarnitine (C3)	Dodecanoylcarnitine (C12)
Thyroid-stimulating hormone (TSH)	Malonylcarnitine (C3-DC)	Dodecenoylcarnitine (C12:1)
17-hydroxyprogesterone (17-OHP)	ButyrylcarnitineþIsobutyrylcarnitine (C4)	Tetradecanoylcarnitine (C14)
**Amino Acids**	Methylmalonylcarnitine (C4-DC)	3-Hydroxytetradecanoylcarnitine (C14-OH)
Alanine (ALA)	IsovalerylcarnitineþMethylbutyrylcarnitine (C5)	Palmitoylcarnitine (C16)
Arginine (ARG)	Tiglylcarnitine (C5:1)	Palmitoleylcarnitine (C16:1)
Isoleucine + Leucine (LEU)	Glutarylcarnitine (C5-DC)	
Methionine (MET)	3-Hydroxyisovalerylcarnitine (C5-OH)	3-Hydroxypalmitoylcarnitine (C16-OH)
Phenylalanine (PHE)	Hexanoylcarnitine (C6)	3-Hydroxypalmitoleylcarnitine (C16:1-OH)
Tyrosine (TYR)	Methylglutarylcarnitine (C6-DC)	Stearoylcarnitine (C18)
Valine (VAL)	Octanoylcarnitine (C8)	Oleoylcarnitine (C18:1)
Tyrosine (TYR)	Octenoylcarnitine (C8:1)	3-Hydroxyoleoylcarnitine (C18:1-OH)
Valine (VAL)	Decanoylcarnitine (C10)	Linoleoylcarnitine (C18:2)

Supplementary Table S2. Variation in Gestational Age Associated with Metabolic Markers and Their Squared and Cubic Terms

Gestational age in weeks	47.3%
Preterm birth (<37 weeks)	32.8%
Very preterm birth (<32 weeks)	31.3%
Number of children	229679

Notes: The unit of analysis is a child. A separate regression model is estimated by regressing each gestational age measure on gender, maternal race, age, marital status, and education, and child's birth year fixed effects first without metabolic markers and then adding them as covariates. Percent of variation in gestational age associated with metabolic markers is the change in R-squared after adding metabolic markers and their squared and cubic terms as covariates. The sample includes children with at least one math test.

Supplementary Table S3. Variation in Gestational Age Associated with Metabolic Markers in Sample Not Matched to School Tests

Gestational age measure	Metabolic markers levels	Metabolic markers levels, squared terms, and cubic terms
Gestational age in weeks	43.3%	47.8%
Preterm birth (<37 weeks)	30.9%	35.6%
Very preterm birth (<32 weeks)	30.3%	36.6%
Number of children	90,532	90,532

Notes: The unit of analysis is a child. A separate regression model is estimated by regressing each measure on gender, maternal race, age, marital status, and education, and child's birth year fixed effects first without metabolic markers and then adding them as covariates. Percent of variation in gestational age associated with metabolic markers is the change in R-squared after adding metabolic markers as covariates. The sample includes children with at least one math test.

Supplementary Table S4. Associations between Gestational Age Measures and School Test Scores Adjusting for Metabolic Markers, Their Squares, and Cubic Terms

	**Math**	**Reading Comprehension**	**Science**
Panel A: Gestational age in weeks	0.46***	0.30***	0.22***
	(0.04)	(0.04)	(0.03)
	[0.38,0.53]	[0.22,0.38]	[0.16,0.29]
Panel B: Preterm or Very Preterm (versus Full term)			
Preterm Birth 32-36 weeks	-0.85***	-0.09	0.30
	(0.23)	(0.24)	(0.20)
	[-1.29,-0.40]	[-0.55,0.37]	[-0.09,0.70]
Very Preterm Birth <32 weeks	-4.03***	-1.81***	-1.91***
	(0.65)	(0.69)	(0.58)
	[-5.31,-2.75]	[-3.16,-0.46]	[-3.04,-0.78]
Number of child-grade observations	973247	973353	909454
Number of unique children	229,679	229,521	222,439

Notes: The Table reports the regression coefficients of gestational age measures in regressions of school tests scores as the dependent variables (outcomes) on gestational age and covariates. Standard errors are in parentheses, and 95% confidence intervals are in brackets. The unit of analysis is a child-grade observation. A separate regression model is estimated for each panel and academic testing domain using ordinary least squares. The reference category in panels B is gestational age≥37 weeks. Standard errors are clustered at the child level. Academic test scores are national percentile rankings. Tests from 1st through 10th grade levels are included for school years 2009-2010 through 2017-2018. All models adjust for gender, maternal race, age, marital status, and education, as well as child's birth year fixed effects, grade fixed effects, school year fixed effects, and metabolic markers. Sample limited to children born between 2002 and 2010. $^{*}p < 0.1$, $^{**}p < 0.05$, $^{***}p < 0.01$.

Supplementary Table S5. Associations between Gestational Age Measures and School Test Scores Excluding Gestational Ages above 41 Weeks and without Adjusting for Metabolic Markers

	Math	Reading Comprehension	Science
Panel A: Gestational age in weeks	0.65***	0.47***	0.40***
	(0.03)	(0.03)	(0.03)
	[0.59,0.70]	[0.42,0.53]	[0.35,0.45]
Panel B: Preterm or Very Preterm (versus Full term)			
Preterm Birth 32-36 weeks	-2.22***	-1.47***	-1.01***
	(0.20)	(0.21)	(0.17)
	[-2.60,-1.83]	[-1.87,-1.07]	[-1.35,-0.67]
Very Preterm Birth <32 weeks	-8.28***	-5.13***	-4.69***
	(0.54)	(0.57)	(0.48)
	[-9.35,-7.22]	[-6.23,-4.02]	[-5.63,-3.76]
Number of child-grade observations	964362	964485	901207
Number of unique children	227,906	227,747	220,742

Notes: The Table reports the regression coefficients of gestational age measures in regressions of school tests scores as the dependent variables (outcomes) on gestational age and covariates. Standard errors are in parentheses, and 95% confidence intervals are in brackets. The sample excludes less than 1 percent of the observations with gestational age at 42-44 weeks. The unit of analysis is a child-grade observation. A separate regression model is estimated for each panel and academic testing domain using ordinary least squares. The reference category in panels B is gestational age≥37 weeks. Standard errors are clustered at the child level. Academic test scores are national percentile rankings. Tests from 1st through 10th grade levels are included for school years 2009-2010 through 2017-2018. All models adjust for gender, maternal race, age, marital status, and education, as well as child's birth year fixed effects, grade fixed effects, and school year fixed effects. Sample limited to children born between 2002 and 2010.

* $p < 0.1$, ** $p < 0.05$, *** $p < 0.01$

Supplementary Table S6. Associations between Gestational Age Measures and School Test Scores Excluding Birth Years 2009 and 2010

	Math	Reading Comprehension	Science
Panel A: Gestational age in weeks	0.62***	0.46***	0.39***
	(0.03)	(0.03)	(0.03)
	[0.56,0.68]	[0.40,0.52]	[0.34,0.44]
Panel B: Preterm or Very Preterm (versus Full term)			
Preterm Birth 32-36 weeks	-2.14***	-1.43***	-0.99***
	(0.20)	(0.21)	(0.18)
	[-2.54,-1.74]	[-1.84,-1.01]	[-1.34,-0.64]
Very Preterm Birth <32 weeks	-8.24***	-5.14***	-4.74***
	(0.56)	(0.58)	(0.49)
	[-9.33,-7.15]	[-6.28,-4.01]	[-5.70,-3.79]
Number of child-grade observations	937027	937467	880290
Number of unique children	201,505	201,468	198,323

Notes: The Table reports the regression coefficients of gestational age measures in regressions of school tests scores as the dependent variables (outcomes) on gestational age and covariates. Standard errors are in parentheses, and 95% confidence intervals are in brackets. The sample excludes birth years 2009 and 2010. The unit of analysis is a child-grade observation. A separate regression model is estimated for each panel and academic testing domain using ordinary least squares. The reference category in panels B is gestational age≥37 weeks. Standard errors are clustered at the child level. Academic test scores are national percentile rankings. Tests from 1st through 10th grade levels are included for school years 2009-2010 through 2017-2018. All models adjust for gender, maternal race, age, marital status, and education, as well as child's birth year fixed effects, grade fixed effects, and school year fixed effects. Sample limited to children born between 2002 and 2010.

Supplementary Table S7. Associations between Gestational Age Measures and School Test Scores Adjusting for 5-Percentile Ranges of Metabolic Markers

	Adjusting for Levels of Metabolic Markers		
	Math	Reading Comprehension	Science
Panel A: Gestational age in weeks	0.43***	0.25***	0.20***
	(0.04)	(0.04)	(0.03)
	[0.35,0.50]	[0.18,0.33]	[0.13,0.27]
Panel B: Preterm or Very Preterm (versus Full term)			
Preterm Birth 32-36 weeks	-0.65***	0.10	0.42**
	(0.23)	(0.24)	(0.20)
	[-1.09,-0.21]	[-0.37,0.56]	[0.02,0.81]
Very Preterm Birth <32 weeks	-3.76***	-1.21*	-1.54***
	(0.63)	(0.66)	(0.56)
	[-4.99,-2.52]	[-2.50,0.09]	[-2.63,-0.45]
Number of child-grade observations	973247	973353	909454
Number of unique children	229,679	229,521	222,439

Notes: The Table reports the regression coefficients of gestational age measures in regressions of school tests scores as the dependent variables (outcomes) on gestational age and covariates. Standard errors are in parentheses, and 95% confidence intervals are in brackets. The unit of analysis is a child-grade observation. A separate regression model is estimated for each panel and academic testing domain using ordinary least squares. The reference category in panels B is gestational age≥37 weeks. Standard errors are clustered at the child level. Academic test scores are national percentile rankings. Tests from 1st through 10th grade levels are included for school years 2009-2010 through 2017-2018. All models adjust for gender, maternal race, age, marital status, and education, as well as child's birth year fixed effects, grade fixed effects, school year fixed effects, and metabolic markers. The model adjusts for metabolic markers by adding 0/1 indicators for the 20 5-percentile ranges of each metabolic marker. Sample limited to children born between 2002 and 2010.

* $p < 0.1$, ** $p < 0.05$, *** $p < 0.01$
